# Neurosyphilis Presenting With Subtle Clinical Features: A Diagnostic and Therapeutic Challenge

**DOI:** 10.1155/crnm/3745783

**Published:** 2026-04-15

**Authors:** Tejash Shahi, Deepti Barakoti, Saurav Ghimire, Manish Subedi, Ram Kumar Sitoula, Prabin Bhattarai, Aakash Koirala

**Affiliations:** ^1^ Department of Medicine, Provincial Hospital Bhadrapur, Jhapa Koshi Province, Nepal

**Keywords:** headache, neurosyphilis, syphilis, vomiting

## Abstract

We report the case of a 36‐year‐old male who presented with an acute onset of headache, vomiting, and feverish sensation with a history of prior unsafe sexual activity. VDRL and TPHA were reactive in both the blood and cerebrospinal fluid. A diagnosis of neurosyphilis was thus made, and we successfully treated the patient with ceftriaxone and doxycycline. With the increasing global trend of syphilis, the involvement of the central nervous system, neurosyphilis, is an increasing concern too. Due to the vague nonspecific symptoms and variable timing of presentation, it presents as a diagnostic challenge. We report this case to emphasize the need to explore the associated risk factors and consider neurosyphilis as a potential diagnosis because timely recognition and early treatment are essential to prevent irreversible neurological consequences.

**Key Clinical Message**
 Acute headache and vomiting can be the only presenting complaint in a patient with neurosyphilis without any other signs. Therefore, sexual history and increased suspicion for neurosyphilis are important for timely diagnosis and treatment.

## 1. Introduction

Neurosyphilis is caused by the invasion of the central nervous system by the spirochete *Treponema pallidum* [1]. While syphilis remains a public health issue with 7.1 million new cases reported worldwide in 2020 [2], neurosyphilis is not as common [3]. Furthermore, neurosyphilis can occur at any stage after infection with *T*. *pallidum*. The clinical presentation ranges from an incidental asymptomatic diagnosis to acute meningeal syphilis, meningovascular syphilis, and late parenchymal syphilis [4]. Due to the variable clinical manifestations and timing of onset, the diagnosis of neurosyphilis remains a challenge. A high index of suspicion is required for diagnosis. Penicillin G is the treatment of choice for neurosyphilis, but ceftriaxone and doxycycline are reasonable alternative therapies [5].

We present a case of a 36‐year‐old male who presented with headache, vomiting, and feverish sensation who demonstrated evidence of neurosyphilis on cerebrospinal fluid (CSF) analysis.

## 2. Case Report

A 36‐year‐old male presented with complaints of headache, vomiting, and feverish sensation for 3 days. The headache was of gradual onset, diffuse, and progressing in severity. He developed three episodes of vomiting from the third day of onset of symptoms. He also complained of a subjective feeling of fever but was not documented at home or at the hospital. The patient had consumed paracetamol at home for the complaints. He denied altered mental status, abnormal body movement, focal neurological deficit, photophobia, or neck stiffness. The patient was employed away from his family and had a history of multiple unsafe sexual exposures between 1 and 2 years prior. He had a medical history of diabetes mellitus of 5 years for which he was taking metformin, linagliptin, and glimepiride. The patient consumed alcohol only socially, did not smoke or consume tobacco, or use recreational drugs.

On examination during presentation, he was well‐oriented and alert. His vitals were blood pressure 140/90 mmHg, heart rate 62 beats per minute, respiratory rate 21 cycles per minute, temperature 97.6 F, and oxygen saturation 98% on room air. Neurological examination was unremarkable with normal higher mental function, cranial nerves, motor, sensory, and cerebellar examinations, and absent meningeal signs. Ophthalmological examination was normal. Examination of the respiratory, cardiovascular, and abdominal systems was normal.

Complete blood count showed a mildly increased total count of 13,500/μL with 81% neutrophils, hemoglobin 14.7 g/dL, and a platelet count of 175,000/μL. Random blood sugar was 302 mg/dL at the time of presentation, and HbA1c was 8.4%. Blood serology showed that venereal disease research laboratory (VDRL) test and *Treponema pallidum* hemagglutination assay (TPHA) test were reactive; HIV, hepatitis B antigen, and hepatitis C antibody were not reactive. Blood VDRL titer was reactive at 1:8 dilution. Cranial CT and MRI are important investigations but could not be performed due to unavailability at our center and financial constraints of the patient. The patient was admitted on the day of presentation, and CSF analysis was performed on the morning (Table 1).

**TABLE 1:** Cerebrospinal fluid analysis.

Test	Result
Total leukocyte count	4 cells/μL
Differential leukocyte count	
Neutrophils	5%
Lymphocyte	95%
Lactate dehydrogenase (LDH)	60 U/L
Protein	40 mg/dL
Sugar	127 mg/dL
Adenosine deaminase (ADA)	18.5 U/L
TPHA	Reactive
VDRL	Reactive 1:8 dilution

CSF analysis showed a normal total leukocyte count and protein level; mildly elevated LDH and increased sugar, reflecting a high serum glucose level. TPHA titer was not available at our center. After confirming the diagnosis, the patient was treated with intravenous ceftriaxone 2 g daily and oral doxycycline 200 mg daily for 2 weeks. This was in accordance with the alternative regimen as aqueous penicillin administration was not feasible for the patient due to the requirement of continuous infusion or frequent dosing needing hospital stay for the entire duration, and procaine penicillin was not used due to the need for daily intramuscular injection. Doxycycline was added to reduce the risk of treatment failure as it is an established antitreponemal drug. Injection glargine was added to optimize his blood sugar level. Pantoprazole was added for gastric ulcer prophylaxis, paracetamol was given for his fever and headache, and ondansetron was given on an as‐needed basis for vomiting. The patient gradually improved. Vomiting had subsided after the administration of ondansetron. The most significant complaint of the patient was headache, which started improving after Day 4 of the antibiotic course and completely subsided by the end of the course. He was stable on discharge. Follow‐up was arranged 2 weeks later and 1 month later. He was free from the disease and achieved glycemic control. Neurological examinations at both visits were normal. Follow‐up visit was advised at 3 months, 6 months, and 1 year. The patient’s partner was also treated with benzathine penicillin.

## 3. Discussion

Neurosyphilis is an infection of the central nervous system by the spirochete *T. pallidum*. The incidence of syphilis is increasing, especially among men who have sex with men and is associated with other sexually transmitted diseases and people living with HIV [2, 6]. Neurosyphilis, although uncommon, is a potential complication. The epidemiology of neurosyphilis is not well known due to the variable time of presentation, unclear or diverse manifestation, and underdiagnosis [7]. A study reported an incidence of 0.47 per 100,000 [8].

Another study has shown that neurosyphilis is reported only in 0.8% of people with syphilis [9]. Neurosyphilis, although rare, has shown an increasing trend with the increasing incidence of syphilis [10].

The spirochete can invade the central nervous system early after infection [10]. The disease, however, can manifest itself at any stage of syphilis: primary, secondary, or tertiary. The condition can be asymptomatic or manifest from early to late years after infection [11]. Our patient presented with nonspecific features of headache and vomiting with a febrile sensation without neurological deficits or meningeal signs. Due to the wide range of manifestations, syphilis has frequently been termed the great imitator [12].

We considered tubercular meningitis, viral meningitis, tropical fever, and metabolic encephalopathy as potential diagnoses. Low levels of ADA made tubercular meningitis less likely, a normal cell count ruled out bacterial meningitis, and negative results for dengue, malaria, typhoid, and scrub typhus ruled out common tropical fevers. Normal metabolic panels excluded metabolic encephalopathies. Our patient had poor glycemic control, and diabetes is an established risk factor for infections, which may have facilitated CNS invasion. The reactivity to VDRL during the screening after a history of unsafe sexual activity provided a clue for syphilis. Thus, we performed CSF analysis.

The CSF analysis of cell count, sugar, and protein is not specific or sensitive for syphilis [13]. CSF VDRL is sensitive as well as specific, whereas CSF‐TPHA has the highest sensitivity but can remain reactive even after successful treatment [14]. Our patient demonstrated reactivity with both CSF VDRL and CSF TPHA tests.

The first‐line regimen is aqueous crystalline penicillin G 3–4 million U every four hours or 18–24 million U per day in continuous infusion for 10–14 days. Ceftriaxone 2 g per day for 10–14 days is a second‐line regimen. Doxycycline is also an alternative therapy and considered an alternative second‐line therapy, especially in patients who have hypersensitivity to penicillin [3, 15]. In our case, ceftriaxone was chosen due to the feasibility of administration and local availability. Ceftriaxone has been shown to have comparable efficacy compared to benzylpenicillin [15, 16]. Doxycycline was added as part of the second‐line regimen. The improvement in clinical symptoms suggests the efficacy of the drugs.

Although our patient developed neurosyphilis, there were no features suggesting complications from the disease. Progressive disease causes endarteritis, necrotic changes, demyelination, and neuronal death, which can lead to paralysis, dementia, and spinal degeneration, which can be permanent, emphasizing the need for early treatment [17].

## 4. Conclusion

Neurosyphilis remains a diagnostic challenge, presenting with atypical, nonspecific, or subtle features. We should develop a suspicion for neurosyphilis in patients presenting with such symptoms due to the increasing global prevalence of syphilis and also inquire about high‐risk behaviors in patients. Early recognition and treatment are important to prevent neurological complications of the disease.

## Author Contributions

All authors were involved in the treatment of the patient.

Tejash Shahi: data curation, conceptualization, original draft writing, reviewing, and editing.

Deepti Barakoti: data curation, original draft writing, reviewing, and editing.

Saurav Ghimire: data curation and original draft writing.

Manish Subedi: conceptualization, original draft supervision, and reviewing.

Ram Kumar Sitoula: data curation and original draft writing.

Prabin Bhattarai: data curation, reviewing, and editing.

Aakash Koirala: Data curation, reviewing, and editing.

## Funding

No funding was required for the publication of this case report.

## Ethics Statement

This study followed the Declaration of Helsinki.

## Consent

Informed expressed consent was obtained from the patient.

## Conflicts of Interest

The authors declare no conflicts of interest.

## Data Availability

The data that support the findings of this study are available from the corresponding author upon reasonable request.
